# Transesophageal vs. transthoracic echocardiography for infective endocarditis: a systematic review and meta-analysis

**DOI:** 10.3389/fcvm.2026.1808304

**Published:** 2026-04-20

**Authors:** Yuan Yang, Wenming Zhang

**Affiliations:** 1 Department of Diagnostic Ultrasound & Echocardiography, Sir Run Run Shaw Hospital, Zhejiang University College of Medicine, Hangzhou, China; 2 Department of Radiology, Sir Run Run Shaw Hospital, Zhejiang University College of Medicine, Hangzhou, China

**Keywords:** diagnostic accuracy, infective endocarditis, meta-analysis, transesophageal echocardiography, transthoracic echocardiography

## Abstract

**Objective:**

This meta-analysis aims to systematically evaluate the diagnostic accuracy of transesophageal echocardiography (TEE) vs. transthoracic echocardiography (TTE) in detecting infective endocarditis (IE).

**Methods:**

A comprehensive computerized search was performed in PubMed, Embase, Web of Science, and Cochrane Library databases to identify relevant English-language diagnostic trials or cohort studies published from inception to September 2025. Two independent researchers conducted literature screening, data extraction, and quality assessment using the Newcastle–Ottawa Scale. Diagnostic accuracy data were extracted or calculated, and pooled sensitivity, specificity, and their 95% confidence intervals (CIs) were determined. A summary receiver operating characteristic (SROC) curve was constructed. Statistical analyses were performed using RevMan 5.3 and Stata 18.0 and related software. Heterogeneity was assessed using the *I^2^* statistic, and publication bias was evaluated using Deeks’ funnel plot asymmetry test.

**Results:**

A total of 13 studies involving 2,765 suspected patients with IE were finally included. A meta-analysis demonstrated that, using TEE as the reference, the pooled sensitivity of TTE was 0.72 (95% CI: 0.55–0.84), and the pooled specificity was 0.72 (95% CI: 0.55–0.85), with significant heterogeneity (sensitivity *I^2^* = 95.96%, specificity *I^2^* = 98.73%). The area under the SROC curve was 0.78 (95% CI: 0.74–0.82), indicating moderate diagnostic performance. Publication bias was detected (*P* = 0.04). Sensitivity analyses confirmed the overall stability of the results, although heterogeneity sources were identified. Subgroup analyses revealed statistically significant heterogeneity in sensitivity among different TTE subgroups (*P* = 0.001), while no significant heterogeneity was observed in specificity subgroups.

**Conclusion:**

TEE remains superior in IE diagnosis. For patients with high clinical suspicion but negative or inconclusive TTE findings, additional TEE examination is recommended to improve diagnostic accuracy and support clinical decision-making.

## Introduction

Infective endocarditis (IE) is a severe infectious disease caused by direct invasion of the endocardium, cardiac valves, or adjacent great vessel endothelium by pathogenic microorganisms. The condition is life-threatening, and delayed diagnosis and treatment can lead to disability or death (1–3). Therefore, early and accurate diagnosis of IE is critical for improving patient prognosis.

Currently, the diagnosis of IE primarily relies on the Modified Duke Criteria, with microbiological evidence and cardiac imaging findings as core components (4). Echocardiography, as the cornerstone for evaluating cardiac structure and function, is the most important imaging tool for IE diagnosis (5). The commonly used clinical modalities are transthoracic echocardiography (TTE) and transesophageal echocardiography (TEE). TTE is often the initial screening method due to its non-invasive nature, convenience, and high reproducibility (6). However, its diagnostic accuracy can be compromised by factors such as body habitus, lung interference, and chest wall structure, potentially limiting the detection rate of characteristic IE lesions such as small vegetations and paravalvular abscesses (7). TEE, by placing the ultrasound probe in the esophagus for close-range cardiac scanning, overcomes many acoustic limitations of TTE and provides superior image resolution (8).

Although TEE demonstrates excellent diagnostic performance, it is a semi-invasive procedure requiring advanced equipment and operator expertise, and patient tolerance is relatively low. Thus, clarifying the diagnostic accuracy differences between TTE and TEE is crucial for establishing rational and efficient imaging workflows in clinical practice. However, existing individual studies have limited sample sizes, and their conclusions regarding the diagnostic value of TTE are inconsistent due to variations in study populations, IE location, ultrasound equipment, and diagnostic criteria (9).

To systematically and quantitatively evaluate the diagnostic accuracy of TTE vs. TEE in IE and provide higher-level evidence for clinical decision-making, this study conducted a meta-analysis of relevant literature published up to September 2025. The aim was to compare pooled sensitivity, specificity, and other diagnostic parameters of TTE using TEE as the reference standard, thereby clarifying their respective clinical utility.

## Materials and methods

### Inclusion criteria

#### Study design

The study design comprised published cohort or cross-sectional studies comparing the diagnostic accuracy of TEE and TTE for IE.

#### Participants

The inclusion criteria were clinically suspected patients with IE, regardless of age, sex, or comorbidities, with a definitive diagnostic reference standard (e.g., Modified Duke Criteria and surgical or autopsy findings).

#### Intervention

The intervention was TTE as the primary diagnostic method.

#### Comparator

The comparator was TEE as the reference diagnostic method.

#### Outcome measures

The outcome measures were studies reporting sufficient data to construct a diagnostic 2 × 2 table (true positives, false positives, false negatives, true negatives).

### Exclusion criteria

The exclusion criteria were the following:

Non-English publications, conference abstracts, case reports, reviews, commentaries, or secondary studies.

Animal studies or studies with unavailable raw data.

Studies not comparing TEE and TTE diagnostic performances or studies with incomplete data for extraction.

Duplicate publications (only the most comprehensive or recent version was included).

### Outcome definitions

#### Sensitivity

Sensitivity was defined as the proportion of TEE-positive (IE-confirmed) patients correctly identified as positive by TTE. Specificity was defined as the proportion of TEE-negative (IE-excluded) patients correctly identified as negative by TTE.

### Search strategy

#### Databases

The databases of PubMed, EMBASE, Web of Science, and the Cochrane Library were searched. Keywords and subject terms were as follows: Primary terms included “Infective Endocarditis,” “Transesophageal Echocardiography,” “Transthoracic Echocardiography,” “Sensitivity,” and “Specificity.” Boolean operators (AND, OR) were used to refine results, example: (“Infective Endocarditis”) AND (“Transesophageal Echocardiography” OR “Transthoracic Echocardiography”) AND (“Sensitivity” OR “Specificity”).

#### Time frame

Articles published from database inception to September 2025 were retrieved to ensure data relevance.

### Study selection and data extraction

Two independent researchers screened studies and extracted data in strict accordance with the PRISMA guidelines, and any discrepancies were resolved by discussion or by a third reviewer. The extracted data were as follows: first author, publication year, country, study design, sample size, baseline patient/lesion characteristics, outcomes, and quality assessment elements. The Newcastle–Ottawa Scale (NOS) was used for quality evaluation.

### Quality assessment

Observational studies (cohort/case–control) were assessed using the NOS across nine domains: Representativeness of suspected IE patients, Clarity of the reference standard, Sample size and case completeness, Standardization of TEE/TTE procedures, Implementation of blinding, Reasonable time intervals between tests, Documentation of key confounders, Completeness of diagnostic data, and Handling of missing data. Each criterion was rated as “met” or “unmet” to determine the suitability of the study for a meta-analysis.

### Statistical analysis

A meta-analysis was performed using RevMan 5.3 and STATA 18.0. Heterogeneity was assessed via the *Q*-test [*Q*-value, degrees of freedom (df), *P*-value] and *I^2^* statistics [with 95% confidence interval (CI)]. Significant heterogeneity was defined as *P* < 0.05 and *I^2^* > 50%. Pooled sensitivity, specificity (with 95% CIs), and summary receiver operating characteristic (SROC) curves [with area under the curve (AUC)] were calculated. Publication bias was evaluated via Deeks' funnel plot (*P* < 0.05 indicated bias). Sensitivity analysis involved sequentially excluding individual studies to verify result stability. Subgroup analyses examined heterogeneity sources by calculating *I^2^*, *P*-values, and between-group differences.

## Results

### Literature search findings

**Figure 1:**
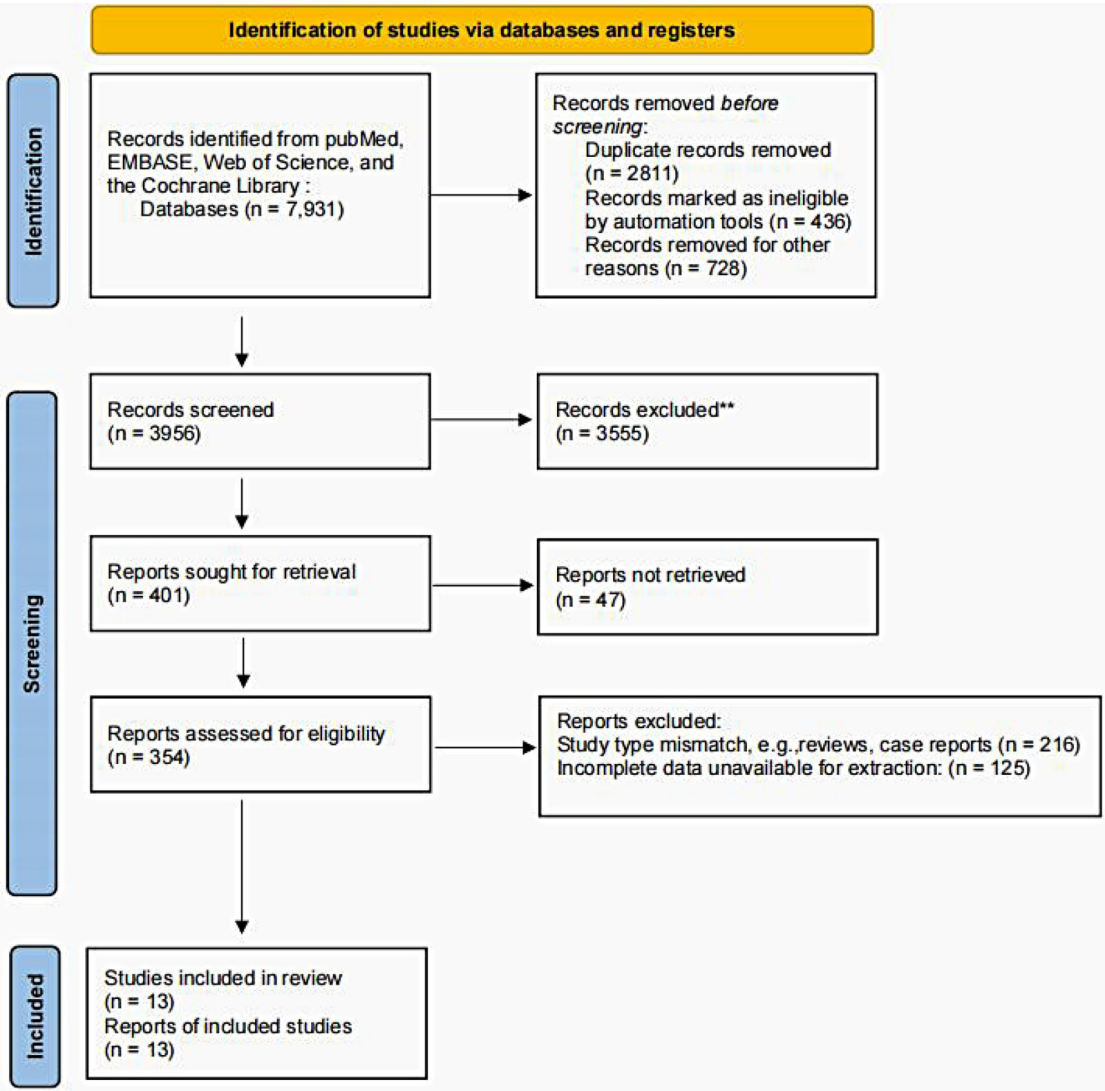
A flowchart of literature selection.

A total of 7,931 records were retrieved. After deduplication, 4,580 articles underwent a full-text review. Thirteen studies met the inclusion criteria (Figure 1).

### Characteristics of included studies

A total of 13 studies were included, involving 2,765 participants, and all of these studies were cohort or controlled studies (Table 1).

**Table 1:** Baseline characteristics of included studies.

Reference	Country	Sample size	Population characteristics	Diagnostic criteria	Imaging technique	Key judgment criteria	TTE 2 × 2 table (tp/fp/tn/fn)	Study design
Barton 2014 (10)	Australia	622	Including prosthetic valves	Modified Duke+TEE	Harmonic TTE and Standard TEE	Only “detected vegetation” as positive	62/129/29/263	Retrospective cohort
Casella 2009 (11)	UK	75	Native valves only	Modified Duke+TEE	Harmonic TTE and Standard TEE	Only “detected vegetation/abscess” as positive	27/15/6/24	Prospective cohort
Irani 1996 (12)	USA	134	Native valves only	Vegetation/abscess+TEE	Standard TTE and Standard TEE	Only “confirmed vegetation/abscess” as positive	41/30/19/44	Retrospective cohort
Jassal 2007 (13)	Canada	36	Native valves only	Modified Duke+TEE	Harmonic TTE and Standard TEE	Only “confirmed vegetation” as positive	16/2/3/14	Prospective cohort
Khan 2022 (14)	USA	213	Native valves only	Modified Duke+typical IE signs+SAB history	Standard TTE and Standard TEE	“Detected vegetation” as positive	38/16/109/8	Retrospective cohort
Kini 2010 (15)	USA	486	Native valves only	Modified Duke+TEE	Standard TTE and Standard TEE	Only “confirmed vegetation/abscess” as positive	60/50/70/217	Retrospective cohort
McDermott 2011 (16)	USA	87	Native valves only	TEE	Standard TTE and Standard TEE	Only “confirmed vegetation” as positive	13/13/4/57	Retrospective cohort
Michałowska 2021 (17)	Poland	44	Including prosthetic valves	Modified Duke+infection-related lesions	Standard TTE and Standard TEE	“Confirmed vegetation/abscess” as positive	15/6/16/10	Retrospective cohort
Pedersen 1991 (18)	USA	24	Including prosthetic valves	Long-term follow-up	Conventional TTE and Standard TEE	Only “identified vegetation/abscess” as positive	2/1/3/8	Prospective cohort
Pfister 2016 (19)	Germany	144	Including prosthetic valves	Modified Duke+clinical/microbiological data	Standard TTE and Standard TEE	Only “confirmed vegetation/abscess” as positive	19/36/1/88	Prospective cohort
San 1993 (20)	Spain	48	Native valves only	TTE and TEE concordance	Conventional TTE and Standard TEE	Only “detected vegetation” as positive	22/0/0/26	Prospective cohort
Shively 1991 (21)	USA	62	Including prosthetic valves	Pathological/clinical criteria	Conventional TTE and Standard TEE	Only “grade 4 definite vegetation” as positive	8/7/1/50	Prospective cohort
Sivak 2016 (22)	USA	790	Native valves only	Modified Duke+TEE	Standard TTE and Standard TEE	Only “suspected vegetation” as positive	68/532/89/101	Retrospective cohort

### Quality assessment

The quality of the included studies was assessed using the NOS. As shown in Table 2, each study was evaluated across nine domains: Representativeness of suspected IE patients, Clarity of diagnostic criteria, Sample size and case completeness, Standardization of TEE/TTE procedures, Implementation of diagnostic blinding, Reasonability of examination intervals, Documentation of key confounders, Completeness of diagnostic data, and Handling of missing data. Each criterion was scored as 1 (fulfilled) or 0 (not fulfilled). The overall quality of the 13 studies was predominantly high quality (nine studies, 69.23%), with moderate quality observed in four studies (30.77%). No low-quality studies were identified. Key limitations were data transparency, bias control, and procedural standardization, but the evidence base was deemed reliable (Table 2).

**Table 2:** Quality assessment of included studies (NOS).

Reference	1	2	3	4	5	6	7	8	9	Scores	Overall of quality	Explanation
Barton 2014 (10)	1	1	1	1	0	1	1	1	1	8	High	No diagnostic blinding (minor bias risk)
Casella 2009 (11)	1	1	0	1	1	1	1	1	0	7	High	Small sample size, partial data coverage, and unclear missing data handling
Irani 1996 (12)	1	1	0	1	1	0	1	1	0	6	Medium	High exclusion rate, unclear examination interval, and opaque missing data handling
Jassal 2007 (13)	1	1	0	1	1	1	1	1	0	7	High	Small sample size, limited data coverage, and unclear missing data handling
Khan 2022 (14)	1	1	1	1	0	1	1	1	0	7	High	No diagnostic blinding and unclear missing data handling
Kini 2010 (15)	1	1	1	1	0	1	1	1	0	7	High	No diagnostic blinding and opaque missing data handling (minor limitations)
McDermott 2011 (16)	1	1	1	1	0	0	1	1	0	6	Medium	No diagnostic blinding, unclear examination interval, and opaque missing data handling
Michałowska 2021 (17)	1	1	1	1	1	1	1	1	0	8	High	Minor limitation due to opaque missing data handling
Pedersen 1991 (18)	1	1	0	1	1	0	1	1	0	6	Medium	Small sample size, imprecise examination interval, and opaque missing data handling (potential bias)
Pfister 2016 (19)	1	1	1	1	1	1	1	1	0	8	High	Minor limitation due to opaque missing data handling
San 1993 (20)	1	1	0	1	1	0	1	1	0	6	Medium	Small sample size, imprecise examination interval, and opaque missing data handling (potential bias)
Shively 1991 (21)	1	1	1	1	1	1	1	1	0	8	High	Minor limitation due to opaque missing data handling
Sivak 2016 (22)	1	1	1	1	1	1	1	1	0	8	High	Minor limitation due to opaque missing data handling

### Meta-analysis results

#### Sensitivity analysis

The sensitivity forest plot revealed variability in the diagnostic sensitivity of TTE relative to TEE. The San et al. study showed extremely high sensitivity (1.00, 95% CI 0.85–1.00), indicating that all TEE-confirmed IE cases were correctly identified by TTE. In contrast, Sivak et al. reported lower sensitivity (0.43, 95% CI 0.35–0.51). The pooled sensitivity was 0.72 (95% CI 0.55–0.84), suggesting that TTE correctly identified ∼72% of IE cases when TEE was the reference. Heterogeneity testing: *Q* = 297.12, df = 12, *P* < 0.001, *I^2^* = 95.96% (94.66–97.26%), which indicated high heterogeneity.

#### Specificity analysis

**Figure 2:**
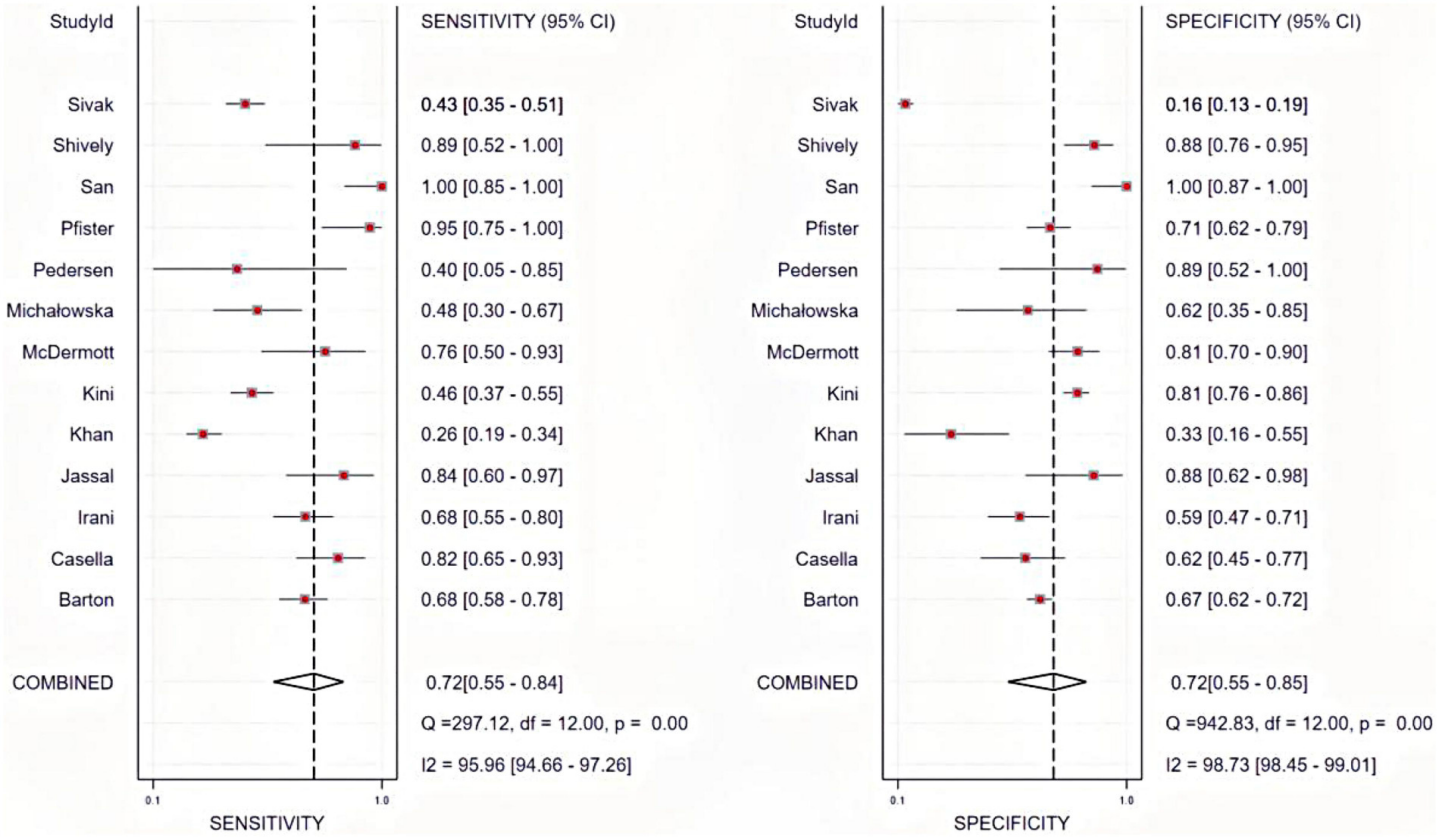
Forest plots of sensitivity and specificity.

The specificity forest plot also demonstrated marked variability. Shively et al. and San et al. reported high specificity (0.98, 95% CI 0.89–1.00; 1.00, 95% CI 0.87–1.00), while Sivak et al. reported low specificity (0.16, 95% CI 0.13–0.19). The pooled specificity was 0.72 (95% CI 0.55–0.85), indicating that TTE correctly excluded ∼72% of non-IE cases when TEE was the gold standard. Heterogeneity testing: *Q* = 942.83, df = 12, *P* *<* 0.001, *I^2^* = 98.73% (98.45–99.01%), which confirmed substantial heterogeneity, warranting further investigation into its sources (Figure 2).

#### SROC curve

The SROC curve demonstrated an AUC of 0.78 (95% CI: 0.74–0.82), indicating favorable overall diagnostic performance (AUC ≥0.75 is generally considered clinically meaningful). The curve exhibited a typical monotonic increase, reflecting the trade-off between sensitivity and specificity (rising specificity was accompanied by a gradually increasing sensitivity). The summary operating point, marked by a red diamond, corresponded to a sensitivity of 0.72 and a specificity of 0.72, consistent with the pooled results from the forest plot. This suggests a balanced diagnostic ability in both “ruling in” patients (sensitivity) and “ruling out” non-patients (specificity).

**Figure 3:**
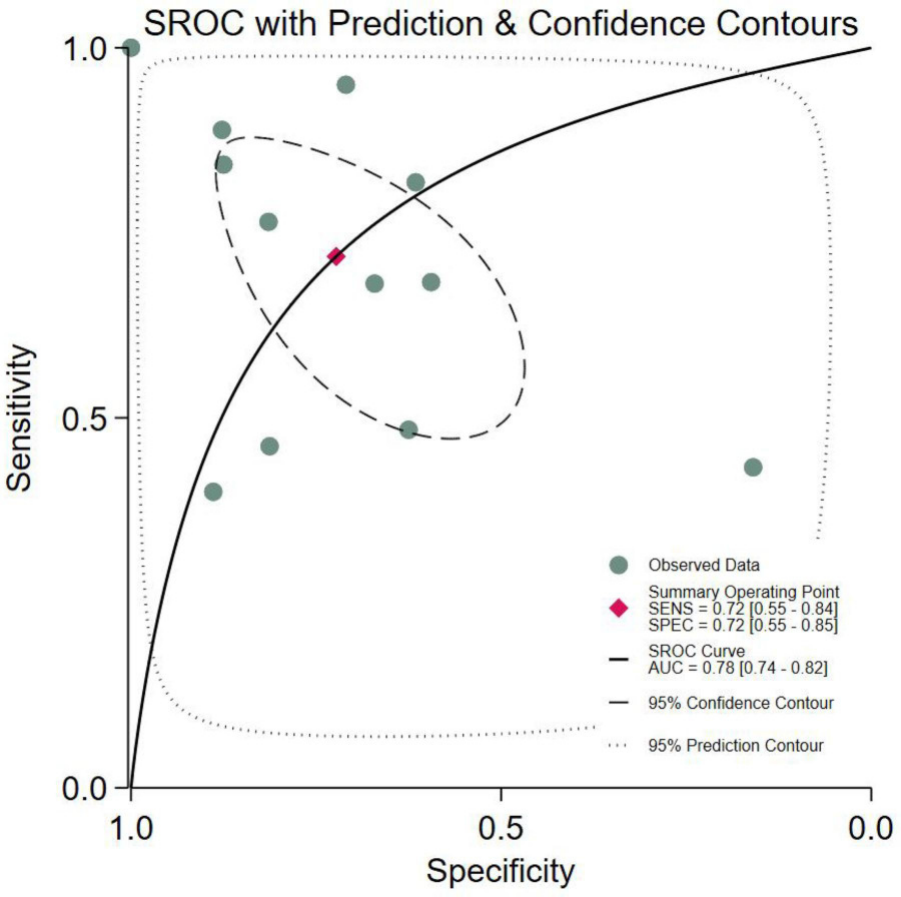
The SROC curve.

The 95% confidence region (dashed line) was narrow, indicating minimal sampling error and robust pooled estimates. In contrast, the 95% prediction region (dotted line) was wide, reflecting substantial heterogeneity across studies and potential variability in future research outcomes. The distribution of observed data points aligned well with the SROC curve, confirming consistency between individual studies and the pooled trend. However, several outliers further underscored interstudy heterogeneity (Figure 3).

#### Publication bias

**Figure 4:**
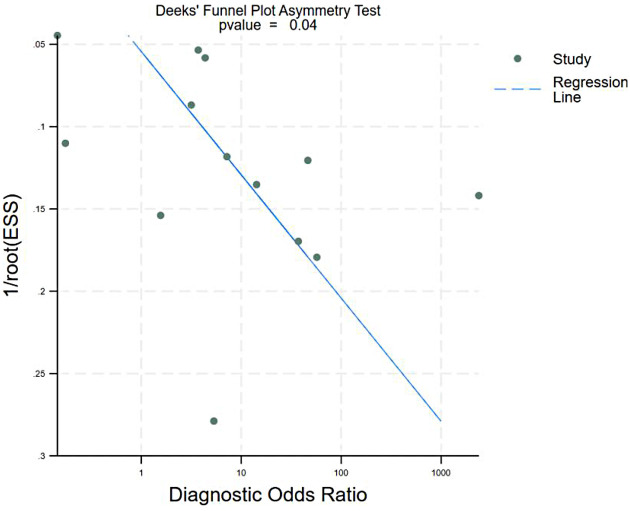
Deeks’ funnel plot for publication bias.

Asymmetrical scatter of study points and significant slope of the regression line (*P* = 0.04) suggested the presence of publication bias in this meta-analysis (Figure 4).

### Sensitivity analysis

The sensitivity analysis demonstrated that the pooled effect sizes (sensitivity and specificity) and heterogeneity exhibited no substantial fluctuations after the exclusion of most studies, indicating overall stability of the meta-analysis results. The exclusion of Khan 2022 led to a reduction in sensitivity I2, while the exclusion of Sivak 2016 significantly reduced specificity I2, suggesting these two studies as potential sources of heterogeneity.

### Subgroup analysis

After excluding San 1993 (a study with high diagnostic sensitivity) in the subgroup analysis, another subgroup analysis based on patient population characteristics revealed the following: Sensitivity: The With artificial valve subgroup (six studies) exhibited high heterogeneity (*I^2^* = 93.6%, *P* < 0.001). The Native valve–only subgroup (six studies) showed low heterogeneity (*I^2^* = 14.5%, *P* = 0.321). The overall pooled analysis (12 studies) demonstrated high heterogeneity (*I^2^* = 87.3%, *P* < 0.001). The between-subgroup heterogeneity (*P* = 0.262) indicated no statistically significant difference in sensitivity heterogeneity across population subgroups. Specificity: The With artificial valve subgroup (six studies) displayed high heterogeneity (*I^2^* = 91.8%, *P* < 0.001). The Native valve–only subgroup (six studies) exhibited moderate heterogeneity (*I^2^* = 59.0%, *P* = 0.032). The overall pooled analysis (12 studies) showed high heterogeneity (*I^2^* = 86.5%, *P* < 0.001). The between-subgroup heterogeneity (*P* = 0.340) suggested no statistically significant difference in specificity heterogeneity across population subgroups.

For the TTE type subgroup analysis, the following results were observed: Sensitivity: The Harmonic-Imaging TTE subgroup (three studies) demonstrated moderate heterogeneity (*I^2^* = 59.6%, *P* = 0.084; borderline significance). The standard TTE subgroup (seven studies) showed no significant heterogeneity (*I^2^* = 0.0%, *P* = 0.645). The traditional TTE subgroup (two studies) exhibited high heterogeneity (*I^2^* = 72.2%, *P* = 0.058; borderline significance). The overall pooled analysis (12 studies) had high heterogeneity (*I^2^* = 87.3%, *P* < 0.001). The between-subgroup heterogeneity (*P* = 0.001) indicated a statistically significant difference in sensitivity heterogeneity among TTE subgroups. Specificity: The Harmonic-Imaging TTE subgroup (three studies) showed moderate heterogeneity (*I^2^* = 51.9%, *P* = 0.125; non-significant). The standard TTE subgroup (seven studies) had high heterogeneity (*I^2^* = 80.7%, *P* < 0.001). The traditional TTE subgroup (two studies) demonstrated very high heterogeneity (*I^2^* = 97.2%, *P* < 0.001). The overall pooled analysis (12 studies) exhibited high heterogeneity (*I^2^* = 86.5%, *P* < 0.001). The between-subgroup heterogeneity (*P* = 0.352) suggested no statistically significant difference in specificity heterogeneity among TTE subgroups.

In the study quality subgroup analysis, the following findings were noted: Sensitivity: The high-quality subgroup (nine studies) had high heterogeneity (*I^2^* = 88.5%, *P* < 0.001). The Moderate-quality subgroup (three studies) exhibited high heterogeneity (*I^2^* = 75.6%, *P* = 0.017). The overall pooled analysis (12 studies) showed high heterogeneity (*I^2^* = 87.3%, *P* < 0.001). The between-subgroup heterogeneity (*P* = 0.334) indicated no statistically significant difference in sensitivity heterogeneity across study quality subgroups. Specificity: The high-quality subgroup (nine studies) displayed high heterogeneity (*I^2^* = 84.0%, *P* < 0.001). The moderate-quality subgroup (three studies) also exhibited high heterogeneity (*I^2^* = 87.1%, *P* < 0.001). The overall pooled analysis (12 studies) demonstrated high heterogeneity (*I^2^* = 86.5%, *P* < 0.001). The between-subgroup heterogeneity (*P* = 0.976) suggested no statistically significant difference in specificity heterogeneity across study quality subgroups (Table 3).

**Table 3:** Subgroup analysis.

Grouping method	Metric type	Subgroup	Number of studies	DerSimonian-Laird method	
				*I^2^*	*P*
Population Characteristics	Sensitivity	With artificial valve	6	93.6	<0.001
		Native-valve only	6	14.5	0.321
		Overall	12	87.3	<0.001
		Heterogeneity between groups, *P* = 0.262			
	Specificity	With artificial valve	6	91.8	<0.001
		Native-valve only	6	59.0	0.032
		Overall	12	86.5	<0.001
		Heterogeneity between groups, *P* = 0.340			
TTE Modality	Sensitivity	Harmonic Imaging TTE	3	59.6	0.084
		Standard TTE	7	0.00	0.645
		Traditional TTE	2	72.2	0.058
		Overall	12	87.3	<0.001
		Heterogeneity between groups, *P* = 0.001			
	Specificity	Harmonic Imaging TTE	3	51.9	0.125
		Standard TTE	7	80.7	<0.001
		Traditional TTE	2	97.2	<0.001
		Overall	12	86.5	<0.001
		Heterogeneity between groups, *P* = 0.352			
Study Quality	Sensitivity	High	9	88.5	<0.001
		Moderate	3	75.6	0.017
		Overall	12	87.3	<0.001
		Heterogeneity between groups, *P* = 0.334			
	Specificity	High	9	84.0	<0.001
		Moderate	3	87.1	<0.001
		Overall	12	86.5	<0.001
		Heterogeneity between groups, *P* = 0.976			

## Discussion

This meta-analysis systematically evaluated the diagnostic accuracy of TTE for IE using TEE as the reference standard. The results demonstrated that TEE maintained significant diagnostic superiority over TTE. Bai et al. first summarized the diagnostic accuracy of TTE vs. TEE for IE (23). In fact, this meta included literature published up to 2016 from North America and Europe, with a total of 11 cohort studies and 1,896 patients with suspected IE enrolled. In our study, we extended the literature inclusion time to September 2025 and finally included 13 high-quality cohort studies with a total of 2,765 participants. The newly added two studies from 2021 to 2025 included the 2021 research by Michałowska et al. from Poland and the 2022 study by Khan et al. from North America (USA), covering the latest diagnostic evidence of IE from both Central Europe and North America. We also supplemented the research evidence of mixed populations of IE patients with native and prosthetic valves that were less discussed in the previous study. In addition, individual newly included studies have drawn controversial conclusions on the diagnostic value of harmonic TTE for IE, which makes the integration of the latest evidence more necessary to clarify the current diagnostic efficacy of TTE. In addition, we adopted multiple subgroup analysis dimensions, including TTE examination modality and study quality stratification, to further explore the potential sources of heterogeneity in the diagnostic accuracy of TTE. This analysis can provide more timely, comprehensive, and targeted medical evidence for the current imaging diagnosis workflow of IE, as well as supplement and improve the relevant research evidence system in this field.

This meta indicated that TTE may serve as an initial screening tool, and its diagnostic performance remains limited. For patients with a high clinical suspicion of IE but negative or inconclusive TTE findings, an additional TEE examination significantly improves diagnostic accuracy, thereby providing more reliable evidence for clinical decision-making. Ultrasonic examination has important clinical guiding significance for the accurate diagnosis of vegetations in infective endocarditis. With the development of interventional therapy technology, percutaneous vegetectomy has become one of the important treatment methods for infective endocarditis (24). The diagnostic accuracy of TEE for determining the size, location, morphology, and mobility of vegetations is significantly higher than that of TTE. TEE can provide accurate imaging evidence for grasping the surgical indications and selecting the surgical path of percutaneous vegetectomy, while TTE, as a screening method, can quickly identify suspected cases of vegetation and provide a reference for judging the timing of early interventional treatment (25). In addition, dynamic ultrasonic monitoring of vegetations can also evaluate the therapeutic effect of percutaneous vegetectomy, judge the residual and recurrence of vegetations after surgery, and provide important evidence for the prognosis evaluation of patients. Therefore, the combined application of TTE and TEE can not only improve the diagnostic accuracy of infective endocarditis but also provide key support for the formulation of individualized interventional treatment plans and prognosis evaluation, and it is an important bridge connecting the diagnosis and treatment of infective endocarditis.

The core findings were derived from 13 eligible studies encompassing 2,765 patients with suspected IE. All included studies were either cohort or case–control designs, conducted across multiple countries, including Australia, the UK, the USA, Canada, Poland, Germany, and Spain. The study populations consisted of patients with both native valves and prosthetic valves. The diagnostic criteria predominantly incorporated the modified Duke criteria supplemented by TEE. The echocardiographic techniques varied, including conventional TTE, standard TTE, and harmonic imaging TTE, ensuring a well-diversified sample that enhances the generalizability of the results.

A pooled analysis revealed that, compared with TEE as the reference, TTE had a pooled sensitivity of 0.72, indicating that TTE correctly identified only ∼72% of IE cases confirmed by TEE, leaving ∼28% of patients potentially missed. The pooled specificity was similarly 0.72, suggesting that TTE correctly ruled out IE in ∼72% of non-IE patients, with a ∼28% false-positive rate. The SROC curve analysis yielded an AUC of 0.78. According to diagnostic test performance standards, an AUC ≥0.75 generally indicates good diagnostic value, implying that TTE demonstrates acceptable but still inferior diagnostic performance compared with TEE, particularly in accurately identifying and excluding IE cases. Bai et al. (23) included 11 cohort studies with 1,896 patients with suspected IE and reported a pooled TTE sensitivity of 0.67 and specificity of 0.76 (with TEE as the reference). By extending literature inclusion to September 2025, our study incorporated 13 high-quality cohort studies, including two key post-2020 studies (14, 17), and revealed that the pooled TTE sensitivity (0.72) and specificity (0.72) showed a slight convergence, which validates the long-term stability of TTE's basic diagnostic efficacy for IE. This convergence also reflects incremental improvements in TTE performance driven by the popularization of standardized ultrasound protocols and harmonic imaging technology over the past decade, as supported by the 2023 study by Damlin et al. (7), which focused on the optimization of TTE diagnostic performance through advanced imaging techniques, and the 2024 study by Ho et al. (8), which demonstrated improved TTE sensitivity (0.81) in low-risk IE patients with native valves when using high-definition harmonic TTE. These findings across different eras and patient cohorts confirm that TTE retains acceptable but inferior diagnostic performance relative to TEE, while also highlighting that the efficacy of TTE can be optimized in specific populations with advanced technology—reinforcing our core conclusion on the complementary roles of TTE and TEE in IE diagnosis.

The substantial heterogeneity observed (I^2^> 95%) may be attributed to multiple factors beyond those explored in the initial subgroup analyses, including study era, patient risk profile, and differences in diagnostic criteria in addition to technical and population-related factors. For prosthetic valves, acoustic shadowing from mechanical components and variations in valve types (mechanical vs. bioprosthetic) significantly impact vegetation detection. In terms of study era, the included studies span from 1991 to 2022, during which ultrasound equipment performance, diagnostic protocols, and clinical understanding of IE have been continuously updated, leading to differences in TTE image acquisition and interpretation. In terms of patient risk profile, the included studies lack a unified stratification of patient risk factors such as underlying heart disease, immune status, and history of invasive procedures, and the different composition of high-risk and low-risk patients in each study may also increase heterogeneity. In addition, minor differences in the diagnostic criteria adopted by each study (e.g., some studies take only “definite vegetation” as the positive criterion, while others include “suspected vegetation”) further contribute to the high heterogeneity of the results.

In addition, interoperator variability in image acquisition and interpretation, the continuous evolution of ultrasound technology over the long time span of the included studies, and the abovementioned uninvestigated factors jointly contribute to the high heterogeneity observed in this meta-analysis, which also explains the limited ability of the initial subgroup analyses to fully clarify the sources of heterogeneity. Recent studies have also conducted in-depth explorations into the sources of heterogeneity in IE diagnosis. A 2023 systematic review by Ivanovic et al. (5) pointed out that differences in the resolution of ultrasound equipment and the seniority and experience of diagnosing physicians are important technical factors contributing to the heterogeneity in TTE diagnosis, which is highly consistent with the analytical conclusions of this study. A 2024 study by Sanchez-Nadales et al. (6) found that regional differences in the study population and variations in the spectrum of underlying diseases significantly affected the manifestations of IE lesions, thereby increasing the heterogeneity of diagnostic results. This is a source of heterogeneity that was not thoroughly explored in this study and provides a new direction for subsequent research. Furthermore, a 2025 study by Montané et al. (9) confirmed that differences in the refinement of IE diagnostic criteria adopted in different studies were also an important cause of heterogeneity in pooled effect sizes, suggesting that future related studies need to unify diagnostic criteria and interpretation specifications. Both sensitivity (*I^2^* = 95.96%) and specificity (*I^2^* = 98.73%) exhibited high heterogeneity, exceeding the 50% threshold, necessitating further investigation into potential sources. Subgroup analyses provided critical insights: Significant intergroup differences in sensitivity heterogeneity were observed (*P* = 0.001). Standard TTE showed no heterogeneity (*I^2^* = 0.00%), whereas harmonic imaging TTE (*I^2^* = 59.6%) and conventional TTE (*I^2^* = 72.2%) demonstrated moderate-to-high heterogeneity. This suggests that TTE technology type is a key contributor—conventional TTE's lower resolution limits its detection of small vegetations and paravalvular abscesses, with variability in device specifications and operator experience exacerbating result discrepancies. Harmonic imaging TTE improves image quality, but differences in equipment models and parameter settings may affect diagnostic consistency. Standard TTE, being more uniformly implemented, yielded more consistent results. The prosthetic-valve subgroup (six studies) exhibited higher sensitivity heterogeneity (*I^2^* = 93.6%) compared with the native-valve subgroup (six studies, *I^2^* = 14.5%). Similarly, specificity heterogeneity was greater in the prosthetic-valve subgroup (*I^2^* = 91.8%) than in the native-valve subgroup (*I^2^* = 59.0%). Although intergroup heterogeneity was not statistically significant (sensitivity *P* = 0.262, specificity *P* = 0.340), these findings reflect the diagnostic challenges posed by prosthetic valves—acoustic shadows from metallic components and suturing rings obscure vegetation/abscess detection, while variations in valve type (mechanical vs. bioprosthetic), implantation duration, and baseline cardiac conditions further amplify heterogeneity. No significant heterogeneity differences were found between high-quality (nine studies) and moderate-quality (three studies) groups (sensitivity *P* = 0.334, specificity *P* = 0.976), with both displaying considerable heterogeneity, suggesting that nonmethodological factors (e.g., clinical or technical variations) predominantly drive variability.

It is necessary to cautiously interpret the clinical significance of the pooled estimates under the condition of high heterogeneity. Although both the pooled sensitivity and specificity of TTE were 0.72 and the AUC of the SROC curve was 0.78, which suggested moderate diagnostic performance, these pooled results are the comprehensive reflection of multiple studies with different study designs, patient populations, and diagnostic standards and therefore cannot be directly applied to all clinical scenarios involving patients with suspected IE. In clinical practice, clinicians should not rely solely on the pooled diagnostic parameters of TTE but should consider them along with a combination of factors such as specific clinical situations (such as the patient's body habitus, whether with prosthetic valves, risk level of IE, etc.), local medical technical conditions, and the experience of ultrasound examiners to comprehensively judge the diagnostic results of TTE and make a rational decision on whether to perform an additional TEE examination. For high-risk IE populations (such as patients with prosthetic valves, a history of endocarditis, and invasive cardiovascular procedures), even if the TTE results are negative or inconclusive, TEE should be performed in a timely manner to avoid missed diagnosis. For low-risk IE populations with typical non-IE clinical manifestations and clear negative TTE results, the necessity of TEE can be comprehensively evaluated to reduce unnecessary invasive examinations.

Publication bias was detected in this study (*P* = 0.04), with Deeks' funnel plot asymmetry indicating potential “positive-result bias”; studies reporting lower sensitivity/specificity may remain unpublished, inflating pooled estimates. The publication bias in this study may lead to the pooled effect size being overestimated to a certain extent; that is, the actual diagnostic sensitivity and specificity of TTE may be slightly lower than 0.72 reported in this study, which also suggests that the rate of missed diagnosis and misdiagnosis of TTE in clinical practice may be slightly higher than the results of this study. However, this publication bias did not alter the core conclusion of this study. The core viewpoint of this study is that TEE still has advantages in the diagnosis of infective endocarditis, and for patients with high clinical suspicion but negative or inconclusive TTE results, an additional TEE examination is required. Even considering the impact of publication bias, the diagnostic accuracy of TTE is still limited and cannot replace the diagnostic value of TEE. At the same time, to reduce the impact of publication bias, we verified the overall stability of the research results through a sensitivity analysis, and we found that the pooled effect size had no significant fluctuation after a single study was excluded, which made up for the deficiencies caused by publication bias to a certain extent. The presence of publication bias suggests that studies with lower diagnostic accuracy estimates may be underrepresented in the literature. This could lead to an overestimation of true diagnostic performance of TTE in clinical practice. The sensitivity analysis showed minimal impact on overall effect sizes upon sequential study exclusion, except for the Khan 2022 (sensitivity *I*^2^ reduction) and Sivak 2016 (specificity *I*^2^ reduction) studies, implying their outsized influence on heterogeneity. Khan 2022 (*n* = 213) incorporated bacteremia history alongside echocardiographic findings, possibly introducing diagnostic threshold variability. Sivak 2016 (*n* = 790, the largest study) defined IE by “possible vegetations,” a less stringent criterion than “definite vegetations/abscesses,” potentially lowering specificity. We ensured strict inclusion of cohort/cross-sectional studies with clear diagnostic criteria, while excluding low-quality literature.

This study has certain limitations. First, the study used TEE as the reference standard for the diagnosis of infective endocarditis, but TEE is not the gold standard for the diagnosis of this disease. The composite clinical standards of modified Duke criteria, surgical/autopsy findings, and clinical adjudication are the generally accepted reference bases. Using TEE as the reference may lead to incorporation bias, which overestimates the diagnostic disadvantages of TTE to a certain extent. The rationality of choosing TEE as the reference standard in this study is that all the 13 included studies took TEE as the core diagnostic basis, and most studies did not fully provide the original 2 × 2 table data based on the composite clinical standards. Restricted by data availability, it is impossible to carry out a unified diagnostic accuracy analysis based on the composite clinical standards; At the same time, TEE is an important means of imaging diagnosis of infective endocarditis in current clinical practice, and the results of this study are more in line with the actual diagnosis and treatment decision-making scenarios of clinicians, with certain clinical practical value.

Comprehensive searches were conducted across PubMed, EMBASE, Web of Science, and the Cochrane Library, spanning database inception to September 2025. Dual-independent study selection, data extraction, and quality assessment using the NOS helped in minimizing bias. However, unexplained residual heterogeneity persists despite subgroup analyses. Future multicenter prospective studies with standardized protocols and meta-regression could clarify confounding effects (e.g., patient demographics and operator expertise).

Publication bias and inadequate data transparency (e.g., unreported blinding and missing confounders) may affect reliability. Therefore, promoting adherence to reporting guidelines and the publication of negative results is essential.

Unaddressed scenarios (prosthetic-valve IE, right-sided IE) and emerging technologies (3D-TTE, transgastric TEE) warrant targeted investigations. Integrating clinical, microbiological, and laboratory markers could optimize diagnostic models.

## Data Availability

The original contributions presented in the study are included in the article/Supplementary Material, further inquiries can be directed to the corresponding author.
